# Association Between Gestational Weeks, Initial Maternal Perception of Fetal Movement, and Individual Interoceptive Differences in Pregnant Women: Cross-Sectional Study

**DOI:** 10.2196/57128

**Published:** 2024-06-26

**Authors:** Miku Furusho, Minami Noda, Yoko Sato, Yoshiko Suetsugu, Seiichi Morokuma

**Affiliations:** 1 Department of Health Sciences Graduate School of Medical Sciences Kyushu University Fukuoka Japan

**Keywords:** fetal movement, gestational weeks, gestation, gestational, heartbeat counting task, interoception, pregnancy, pregnant, maternal, fetus, fetal, association, associations, correlation, correlations, obstetric, obstetrics, interoceptive, perception, perceptions, awareness, sense, sensing, senses, internal stimulus, internal stimuli

## Abstract

**Background:**

Interoception encompasses the conscious awareness of homeostasis in the body. Given that fetal movement awareness is a component of interoception in pregnant women, the timing of initial detection of fetal movement may indicate individual differences in interoceptive sensitivity.

**Objective:**

The aim of this study is to determine whether the association between the gestational week of initial movement awareness and interoception can be a convenient evaluation index for interoception in pregnant women.

**Methods:**

A cross-sectional study was conducted among 32 pregnant women aged 20 years or older at 22-29 weeks of gestation with stable hemodynamics in the Obstetric Outpatient Department. Interoception was assessed using the heartbeat-counting task, with gestational weeks at the first awareness of fetal movement recorded via a questionnaire. Spearman rank correlation was used to compare the gestational weeks at the first awareness of fetal movement and heartbeat-counting task scores.

**Results:**

A significant negative correlation was found between the gestational weeks at the first fetal movement awareness and heartbeat-counting task performance among all participants (*r*=–0.43, *P*=.01) and among primiparous women (*r*=–0.53, *P*=.03) but not among multiparous women.

**Conclusions:**

Individual differences in interoception appear to correlate with the differences observed in the timing of the first awareness of fetal movement.

## Introduction

A pregnant woman typically first senses fetal movement at approximately 18-20 weeks of gestation in primipara and approximately 16-18 weeks in multipara. However, there is variability in the gestational week when this awareness occurs, with some experiencing it earlier or later [1,2]. The factors contributing to these variations remain unknown. Interestingly, this awareness tends to occur at approximately 16 weeks or after 20 weeks of gestation. Fetal movements begin in the eighth week of pregnancy, initially subtle and imperceptible to pregnant women. In the absence of maternal or fetal complications, differences in fetal development up to 20 weeks of gestation are minimal [3]. Therefore, fetal development is unlikely to influence a pregnant woman’s initial awareness of fetal movement.

Recently, interoception has attracted attention in the fields of psychosomatic medicine and psychology [4]. The term “interoception” was first coined by the British physiologist Sherrington [5] in 1906. It refers to awareness related to changes inside the body, such as the movement of the heart and internal organs, signifying a crucial aspect of overall bodily homeostasis [6]. However, the measurement of interoception is complicated by the need to use questionnaires or a heartbeat-counting task.

Considering that the awareness of fetal movement is considered a component of interoception in pregnant women, variations in the gestational weeks at which initial detection occurs may indicate individual interoceptive disparities. These deviations may lead to mental and physical illnesses, such as mood and metabolic disorders [7]. During pregnancy, mood disorders related to anxiety and depression often develop. However, there is no easy way to detect mental problems in pregnant women [8].

Therefore, establishing the correlation between the gestational week of first fetal movement awareness and interoception could serve as an evaluation index for interoception in pregnant women and may predict mental problems. However, to our knowledge, no previous study has examined the association between interoception and the gestational week at the first fetal movement awareness in pregnant women. Thus, in this study, we aimed to clarify this noteworthy association.

## Methods

### Study Design

A cross-sectional study was conducted among the recruited 32 pregnant women aged 20 years or older at 22-29 weeks of gestation with stable hemodynamics in the Obstetric Outpatient Department of Kyushu University Hospital. The study was conducted between July and September 2019. Mothers with obvious fetal morphological abnormalities or maternal complications were excluded from recruitment.

### Procedure

The data sampling was conducted in a quiet outpatient private room to avoid outside noise, as described in the previous study [9]. First, a wearable heart rate sensor (WHS-1, Union Tool Co.) was attached to the left precordial area, and the participants were allowed to sit and rest for 5 minutes. Then, the heartbeat-counting task was conducted.

### Clinical Characteristics

The pregnant women’s health status and personal information (including age, gestational period in weeks, educational background, past and current medical history, obstetric history, height, weight, drinking status, smoking status, fertility treatment status, employment status, and financial status) were obtained from the medical records and questionnaires. BMI was calculated using the above data.

### Measurement of Interoception

There are different methods for measuring interoception. In the heartbeat-tracking task [10], the participant is asked to press a button on the experimental device synchronous with their heartbeat. In the heartbeat discrimination task [11], the participant is asked to discriminate a sound that matches the heartbeat from a sound that deviates from the heartbeat. In the heartbeat-counting task [10], the number of heartbeats felt by the participant is compared with the actual number of heartbeats measured using an electrocardiogram (ECG) within a certain period. In this study, we used the heartbeat-counting task developed by Schandry [12] to measure interoception, which can be performed in an outpatient setting.

For the measurement procedure, the participants were asked to sit on a chair in the laboratory and were instructed not to touch their bodies to avoid obtaining cues by touching their pulse points. In this state, the participants were asked to count the number of times they felt a heartbeat at 3 intervals of 25, 35, and 45 seconds and to complete a preprepared form after each interval. The absolute value of the difference between the participants’ reported heartbeats and the actual ECG-measured heartbeats during each interval was calculated. This absolute difference was divided by the actual number of heartbeats separately for each of the 3 intervals to obtain the ratio of deviation in heartbeats. This value was subtracted from 1, and the mean of all 3 intervals was calculated. This value was used as the heartbeat-counting task score. The heartbeat-counting task score ranged from 0 to 1. The closer the score was to 1, the more accurately the participant felt her heartbeat [4,12].

### Statistical Analysis

Descriptive statistics were calculated, and the Mann-Whitney *U* and Kruskal-Wallis tests were used to compare the data between the groups. Spearman rank correlation was used to compare the gestational weeks at the first awareness of fetal movement and the heartbeat-counting task scores. All analyses were performed using SPSS (version 27; IBM Corp). The significance level was set at 5% or *P*<.05.

The sample size calculation was performed using G*Power 3.1.9.7 [13]. Assuming a 2-tailed test for the population correlation coefficient with an expected correlation coefficient of 0.5, a significance level of 5%, and a power of 80%, the required sample size was calculated to be 26 cases.

### Ethical Considerations

The Ethics Committee of Kyushu University Hospital (No. 22071-00) approved this study, and all participants provided written informed consent. All the research procedures were conducted following the tenets of the Declaration of Helsinki.

Information on the participants and the data used in this study were collected from a previous report [9], which showed an association between interoception and anxiety. Additionally, data regarding the gestational week at the first awareness of fetal movements in pregnant women were added. Permission was obtained from the authors of the previous study.

## Results

Among the 32 participants, the mean gestational week at the first fetal movement awareness was 18.3 (SD 2.6). Table 1 compares the gestational weeks at the first fetal movement awareness based on the participants’ characteristics. There were no significant differences in the gestational weeks of the first fetal movement based on participant characteristics (all *P*>.05).

**Figure 1:**
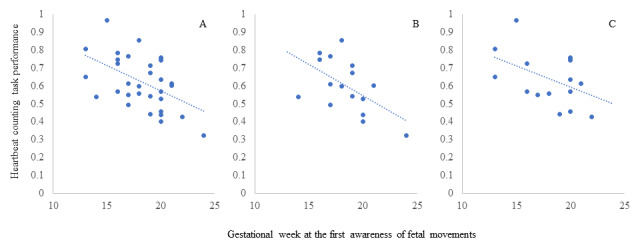
Correlation between the gestational weeks at the first fetal movement awareness and heartbeat-counting task performance: (A) all participants, (B) primiparous women, and (C) multiparous women.

A significant negative correlation (*r*=–0.43, *P*=.01) was found between the gestational weeks at the first fetal movement awareness and heartbeat-counting task performance among all the participants (Figure 1A).

In primiparous women, a significant negative correlation (*r*=–0.53, *P*=.03) was found between the gestational weeks at initial fetal movement awareness and the heartbeat-counting task performance (Figure 1B). However, for multiparous women, there was no significant association between the gestational weeks at initial fetal movement awareness and heartbeat-counting task performance (*r*=–0.35, *P*=.18; Figure 1C).

**Table 1:** Participant characteristics and gestational weeks at the first awareness of fetal movement (N=32).

Characteristics			Participant, n (%)		GWs^a^ at the first awareness of FM^b^, mean (SD)		*P* value	
**Mother’s age (years)**								.39
	<35	21 (65.6)		18.5 (2.8)				
	≥35	11 (34.4)		17.8 (2.2)				
**Parity**								.32
	Primipara	16 (50)		18.4 (2.4)				
	Multipara	16 (50)		18.1 (2.8)				
**BMI**								.44^c^
	<18.5	4 (12.5)		17.5 (3.0)				
	18.5-25	24 (75)		18.1 (2.4)				
	≥25	4 (12.5)		20.3 (2.6)				
**Fertility treatment during this pregnancy**								.55
	No	24 (75)		18.1 (2.8)				
	Yes	8 (25)		18.9 (1.7)				
**Employment status**								.74
	Working	18 (56.3)		18.2 (2.9)				
	Not working	14 (43.8)		18.4 (2.2)				
**Smoking**								.14
	Previously smoked	3 (9)		20.0 (0.0)				
	No smoking	29 (91)		18.1 (2.6)				

^a^GW: gestational week.

^b^FM: fetal movement.

^c^Kruskal-Wallis test.

## Discussion

### Principal Findings

We found a significant association between the gestational week at initial fetal movement awareness and performance on the heartbeat-counting task. In terms of parity, the association between the gestational week at the first awareness of fetal movement and heartbeat-counting task performance was found in primiparous women but not in multiparous women. Although the reasons for the individual differences in fetal movement awareness remain unclear, our results indicate a link between these differences and individual variations in interoception.

The average number of weeks at which fetal movement was first detected in the participants of this study was 18.3 weeks. *Williams Obstetrics* estimated it to be around 18-20 weeks for primiparous women and around 16-18 weeks for multiparous women [1]. Other studies reported that most pregnant women experience the onset of fetal movement at 16-20 weeks [2,14,15]. Therefore, we posit that the number of weeks at which fetal movements are first noticed in the participants of this study is approximately the same as the average number of weeks.

Primiparous women have difficulty distinguishing fetal movements from stomach and bowel movements, as fetal movements represent an unfamiliar sensation to them [14,15]. Regarding awareness of fetal movements, Tuffnell et al [16] stated that awareness of fetal movements is caused by pressure on the pregnant woman’s body wall structure. Interoceptive sensations are sensations related to the internal environment of the body and its changes, such as the heartbeat, and internal organs, such as the stomach and intestines. Pressure on body wall structures is also a part of interoceptive sensation. Furthermore, the accuracy of interoceptive sensation is a value that objectively measures how accurately a person can grasp the internal situation through the senses [17]. Therefore, it is thought that the more accurately a person can detect fetal movements, the more accurate is their interoceptive sense.

Few studies have explored interoception in pregnant women, highlighting the need for further investigation in this area. Furthermore, as it has been reported that deviations in interoception may lead to mental and physical illnesses, such as mood and metabolic disorders [6], it is necessary to examine whether the gestational week at initial fetal movement awareness correlates with maternal mental characteristics and challenges during the peri- and postnatal periods.

### Limitations

The generalizability of this study’s findings may be limited when restricted to primiparous or multiparous women because of the small sample size. Moreover, the method used, which relied on pregnant women recalling and describing the gestational week of their first fetal movement experience, introduces the possibility of recall bias, which cannot be excluded.

### Conclusions

Individual differences in interoception are related to individual differences in the first awareness of fetal movement and can be a crucial evaluation index for interoception in pregnant women.

## Abbreviations

ECG: electrocardiogram
